# The Change of Perinatal Mortality Over Three Decades in a Reference Centre in the Aegean Region: Neonatal Mortality has decreased but Foetal Mortality Remains Unchanged

**DOI:** 10.4274/balkanmedj.2016.0870

**Published:** 2017-12-01

**Authors:** Nilgün Kültürsay, Niyazi Aşkar, Demet Terek, Ahmet Özgür Yeniel, Özge Altun Köroğlu, Mehmet Yalaz, Ferda Özkınay, Mete Akısü

**Affiliations:** 1 Department of Pediatrics Division of Neonatology, Ege University School of Medicine, İzmir, Turkey; 2 Department of Obstetrics and Gynecology, Ege University School of Medicine, İzmir, Turkey; 3 Department of Pediatrics Division of Genetics, Ege University School of Medicine, İzmir, Turkey

**Keywords:** Perinatal mortality, neonatal mortality, foetal mortality, premature birth

## Abstract

**Background:**

Perinatal, foetal and neonatal mortality statistics are important to show the development of a health care system in a country. However, in our country there are very few national and regional data about the changing pattern of perinatal neonatal mortality along with the development of new technologies in this area.

**Aims:**

Evaluation of the changes in mortality rates and the causes of perinatal and neonatal deaths within years in a perinatal reference centre which serves a high-risk population.

**Study Design:**

Cross-sectional retrospective study.

**Methods:**

The perinatal, neonatal and foetal mortality rates in the years 1979-1980 (1^st^ time point) and 1988-1989 (2nd time point) were compared with the year 2008 (3rd time point). The causes of mortality were assessed by Wigglesworth classification and death reports. The neonatal mortality in the neonatal intensive care unit was also calculated.

**Results:**

Foetal mortality rates were 44/1000, 31.4/1000 and 41.75/1000 births, perinatal mortality rates were 35.6/1000, 18.8/1000 and 9/1000 births, and neonatal mortality rates were 35.6/1000, 18.8/1000 and 9/1000 live births for the three study time points, respectively. The mortality rate in neonatal intensive care unit decreased consistently from 33%, to 22.6% and 10%, respectively, together with decreasing neonatal mortality rates. The causes of perinatal deaths were foetal death 85%, immaturity 4%, and lethal congenital malformations 8% according to Wigglesworth classification in 2008, showing the high impact of foetal deaths on this high perinatal mortality rate. Infectious causes of neonatal deaths decreased but congenital anomalies increased in the last decades.

**Conclusion:**

Although neonatal mortality rate decreased significantly; foetal mortality rate has stayed unchanged since the late eighties. In order to decrease foetal and perinatal mortality rates more efficiently, reducing consanguineous marriages and providing better antenatal care for high risk pregnancies are needed.

Perinatal, foetal and neonatal mortality statistics are indicators of the efficiency of a health care system in a country (1,2,3). These statistics must be carefully followed and evaluated and the causes of foetal and neonatal deaths must be clarified in order to take necessary preventive measures to decrease perinatal mortality rates.

There is a great variation between countries; however, for international comparison, the perinatal period is defined as “22 completed weeks of gestation and the first seven postnatal days” by World Health Organization (WHO) (3). However, the perinatal period definition starts from 20 completed weeks of gestation, according to National Centre for Health Statistics of the Centres for Disease Control and Prevention definition-3 used in the USA and some other countries (4).

The fourth goal of the Millennium Development Goals accepted by world leaders with the pioneering efforts of the WHO and UNICEF, was to decrease infant mortality by 2/3 from 1990 up to 2015. Unfortunately, despite great efforts, worldwide neonatal mortality rate (NMR) could be decreased from 33/1000 live births to 22/1000 live births, with a reduction of 40% (5). The reduction in foetal mortality rate (FMR) (14.5%) was much smaller, decreasing from 22.1/1000 births in 1995 to 18.9/1000 births in 2009.

Africa has the highest rank for stillbirths and neonatal deaths, being 10-fold higher than that of high income countries. More than 50% of all perinatal-neonatal deaths occur in 5 countries (India, Nigeria, Pakistan, China and Congo) (6).

In 2009, 76.2% of foetal deaths occurred in South Asia and Sub-Saharan Africa, 55% of which occurred in rural areas of these countries. Half of these deaths are due to complications related to delivery (7). In our country, in a small number of national or institutional studies, perinatal mortality rate is given between 16.9/1000 and 125/1000. Moreover, since the definitions of perinatal mortality differs between studies, it is difficult to make comparisons.

The aim of the present study is to evaluate the changes in mortality rates and the causes of perinatal and neonatal deaths within years in a perinatal reference centre, which serves to a high-risk population, in order to get information to help improvements in perinatal care.

## MATERIALS AND METHODS

In the present study, data of infants born after the 20^th^ gestational week and/or with a birth weight of over 500 grams in the department of obstetrics and gynaecology from 3 time point over 30 years were recorded. The first time point was between 1979 and 1980; the second time point was between 1988 and 1989; and the third time point was between January 1st and December 31^st^, 2008. Perinatal mortality rate (PMR), FMR and NMR were calculated for each time point. Mortality rates of the neonatal intensive care unit (NICU) were also assessed for each time point. All data about mortality rates obtained for each time point were compared.

The time point of 1979-1980 was the first time point, correlating with the poorest perinatal facilities in our hospital and in the region. In the second time point of 1988-1989 the infants were still cared for in a NICU with poor facilities, with no ventilatory care and with a very high patient/nurse ratio in NICU. Antenatal surveillance with ultrasound and follow-up of high risk pregnancies were started during this period. In 1990, the NICU was modernised by a trained neonatologist* and in 1995 the first surfactant administrations and invasive ventilator strategies were initiated. The infection control guidelines were strictly operated after the year 2000, and patient/nurse ratios improved. During the years 2000-2008, many neonatologists were trained in the unit, and nurses were better educated before and during their service. Antenatal diagnostic facilities also improved. The mortality numbers in the year 2008 are thought to show the cumulative effects of all of these efforts. Therefore, we decided to analyse the causes of perinatal deaths for the first two time points with similar poor facilities together, comparing them to the last time point, with improved facilities and better scientific knowledge and experience in neonatal-perinatal care. Ethical committee approval was obtained from Ege University with the ethical committee decision number 11-12.2/4.

### Definitions

*Live birth* is defined as a newborn who is breathing, has brachial arterial pulsatility or has extremity movements.

*Foetal death* is defined as a foetus without any signs of activity after 20 weeks of gestation or a birth weight of over 500 grams.

*Perinatal period* denotes the first seven days postnatally and the last 20 weeks of gestation.

*Foetal mortality rate* is calculated as the number foetal deaths over 20 weeks of gestation per 1000 births (number of foetal deaths × 1000): number of total births.

*Neonatal mortality rate* is calculated as the number of neonatal deaths in the first 28 days of life per 1000 live births (number of newborn deaths × 1000): Number of live births.

*Early neonatal mortality rate* is the number of neonatal deaths in the first 7 days of life per 1000 live births (number of early newborn deaths × 1000): Number of live births.

*Late neonatal mortality rate* is the number of neonatal deaths occurring after the seventh day to 28 days of life per 1000 live births (number of late newborn deaths × 1000): Number of live births.

PMR is the sum of foetal and early neonatal deaths in 1000 births (foetal deaths + early neonatal deaths) × 1000: Number of total births.

Respiratory distress syndrome (RDS) was diagnosed clinically with early respiratory distress manifested as cyanosis, grunting, retractions and tachypnoea. The diagnosis was confirmed with blood gas analysis and chest X-ray with a classical “ground glass” appearance and air bronchograms (8).

Asphyxia means a critical shortage of oxygen during labour and delivery sufficient to produce lactic acidosis and delay the onset of respiration. Criteria for asphyxia involve a low Apgar score, low pH and increased base deficit (9).

Sepsis was defined as documentation of infection with a serious systemic illness in which non-infectious explanations for the abnormal pathophysiological state are excluded or unlikely. By definition, in early clinical sepsis, clinical signs appear in the first 5 days and in late sepsis >5 days (10).

Wigglesworth classification is commonly used to classify perinatal mortality causes in primary health care centres without any necessity for post-mortem investigation (11). The causes of perinatal mortality according to Wigglesworth classification are grouped as foetal deaths, lethal malformations, immaturity, perinatal hypoxia, special causes, infections, and others.

Wigglesworth analysis for the causes of perinatal deaths could only be prospectively performed for the deaths in 2008. The causes of neonatal deaths were also recorded according to the death reports.

## RESULTS

**FIG. 1.**
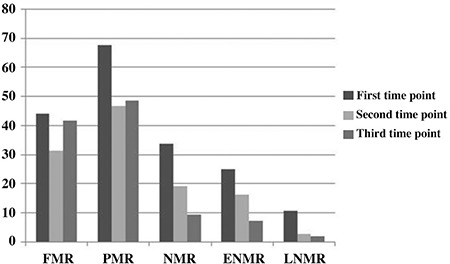
Foetal, perinatal, neonatal, and early neonatal vs. late neonatal mortality rates (/1000) for three different time points. FMR: foetal mortality rate; PMR: perinatal mortality rate; NMR: neonatal mortality rate; ENMR: early neonatal mortality rates; LNMR: late neonatal mortality rates

Total number of births, live births and mortality rates in different study time points are given in Table 1. FMR was calculated as 44/1000, 31.4/1000 and 41.75/1000 for each study time point, respectively (Table 1 and Figure 1). PMR decreased from 69/1000 to 47.6/1000 in the second time point and stabilised as 48.9/1000 in the third time point.

The causes of perinatal mortality in 2008 were classified as foetal death (85%), immaturity (4%) and lethal malformations (8%) according to Wigglesworth classification.

NMR was calculated as 35.6/1000, 18.8/1000 and 9/1000 for each study time point in order. The early NMR was determined as 25/1000, 16.2/1000 and 7.2/1000 for each study time point. The late NMR was 10.6/1000, 2.63/1000 and 1.8/1000 for each study time point in order (Table 1, Figure 1).

Table 2 shows the causes of neonatal mortality.

The mortality rate in NICU decreased consistently as 33%, 22.6% and 10% at the study time points, with the technological advancements and scientific improvements in neonatal care. The total number of newborns cared for in our NICU was 1000 in 1979-1980, 600 in 1988-1989, and 250 in 2008. In 1979-1980, 30% of patients were outborn, 25% in 1988-1989 and 20% in 2008.

## DISCUSSION

Our results showed that foetal deaths and perinatal mortality have not changed much in our perinatal reference centre in western Anatolia since the late 1980s. However, NICU mortality rates together with NMR were reduced with the improved neonatal care, increasing the survival chance of sick infants.

Better antenatal care, appropriate management of deliveries and improved conditions of the NICUs are important parameters associated with low PMRs. PMR is closely correlated with the developmental level of countries and is under 10 per 1000 live births in developed countries. It is reported to be as high as 62/1000 in Africa and 50/1000 in Asia in WHO reports, and as low as 10/1000 in developed countries (5).

In limited numbers of regional and nationwide studies in Turkey, PMR is reported in a range from 16.9/1000 to 125/1000 (12,13,14,15,16,17,18). There is a wide difference between the definitions of the perinatal period, which makes the comparison of these studies difficult.

Demographic and Health Surveys of University Institute of Population Studies reported a high PMR of 43/1000 in 1993 and a decrease as 19/1000 in 2008 and 11/1000 in 2013 in Turkey (19,20). The Turkish Ministry of Health reported a PMR of 19/1000 in 2008 and 11/1000 in 2013. The NMRs reported for the same periods were 13/1000 and 7/1000 (21).

However, the collection of these data may not be suitable for generalisation, especially for higher level perinatal centres. Actually, data collected in a more controlled manner in local studies at university hospitals do not follow the same trajectory, showing a limited decrease in PMR due to high FMR despite decreased early NMR. Our data over a 30-year period are consistent with these findings. This variability seen in studies from university hospitals may also originate from the fact that these are the centres where the highest risk deliveries take place.

In the multicentre study of Turkish Neonatal Society, evaluating deliveries over 22 weeks of gestation and over 500 grams of birth weight from 29 large state hospitals and university hospitals, PMR was reported as 34.9/1000. FMR was 18/1000 and early NMR was 17/1000 in 1999 (22). The highest PMRs were observed in the rural and semi-urban areas with a lower socioeconomic status. In local studies, Madazlı (23) reported a PMR of 43.2/1000, FMR of 24/1000, and early NMR of 19/1000 between 1986 and 1992. Yalınkaya et al. (24) reported a PMR of 125/1000, FMR of 45/1000 and NMR of 80/1000 between 2001 and 2002. Perinatal Mortality Research Group (17) reported a PMR of 33/1000 in 1998-1999 and 16/1000 in 2000-2001. Foetal deaths constituted 61% and 58% of perinatal deaths consequently (17). Aygün et al. (25) reported high PMRs of 87.5/1000, 73.2/1000 and 73/1000 in the years 2004, 2005 and 2006, respectively, from a university hospital in northern Turkey. FMRs were reported as 65.9/1000, 47.4/1000 and 53/1000 whereas early NMRs were 21.5/1000, 25.8/1000 and 20/1000 in order. Early NMR decreased by 50% in the total study period and half of the perinatal mortality was due to foetal deaths. Ecevit et al. (15) reported a decrease in perinatal mortality due to decreased early NMR in the largest maternity hospital of middle Anatolia whereas FMR stayed high comparing data of 1998 and 2009. These local data show that perinatal mortality began to decrease in recent decades in most regions of Turkey due to the improvements in neonatal care, but foetal mortality is still high.

Our recent data of 2008 showed that PMR (48.9/1000) and FMR (41.75/1000) are higher than the national data, although our NMR (10/1000) is lower than the reported national NMR of 13/1000 for 2008 (21). The perinatology unit of our university is the main reference centre for the region for high-risk pregnancies, which is the reason for the high FMRs. In accordance with the reduced NMR, mortality rates in our NICU decreased during the study time points by 33%, 22.6% and 10%, respectively. Improved neonatal care due to new techniques and scientific improvements in the care of preterm and high risk infants helped to reduce the mortality rates.

PMRs are statistical data and do not include information about the aetiology of mortality. In order to take preventive measures, the causes of perinatal mortality should also be classified. The problem in the classification of perinatal deaths is that although a single aetiology is given for the death, the cause of the death is mostly multifactorial. Wigglesworth classification is commonly used to classify perinatal mortality cases in primary health care centres without any necessity for post-mortem investigation (8). The shortcoming of the Wigglesworth classification is that it is not possible to identify the real cause of mortality. Post-mortem autopsy should be performed to clarify the real cause of death and identify the congenital anomalies. The information obtained by post-mortem autopsy, and genetic testing will help to calculate the risk of recurrence in the successive pregnancies (26).

The main causes of perinatal mortality were foetal death (85%), immaturity (4%) and lethal malformations (8%) in 2008. In a previous study, we investigated 1562 foetal and 610 neonatal autopsies during the period of 1967-1989, and showed that the main causes of foetal deaths were perinatal asphyxia (57%), congenital anomalies (24%) and intrauterine foetal infections (10%) in our centre. The main causes of neonatal deaths were intrauterine and neonatal infections (26%), RDS (31%), immaturity (18%) and congenital anomalies (10%), among others (27).

In the recent study, the causes of neonatal deaths were RDS/immaturity (49%) at the first rank followed by neonatal sepsis (26%) and congenital anomalies (10%) in the former two study time points; however, congenital anomalies (25%) were the second highest cause after immaturity/RDS (60%) in 2008. Asphyxia (5%) and respiratory problems other than RDS (5%) were the other leading causes (Table 2).

In the multicentre study of the Turkish Neonatal Society in 1999, foetal deaths (42.7%) were the most frequent cause of perinatal death, followed by prematurity (26%) and lethal congenital malformations (13.2%), according to Wigglesworth classification (22). The Perinatal Mortality Study Group (12) reported the causes of perinatal deaths to be prematurity (29%), congenital malformation (26%) and macerated foetal death (22%) in the period of 1998-1999. This group indicated that the most common cause of perinatal mortality changed from prematurity to congenital anomalies in the period from 2001-2006 (13).

In 2014, UNICEF reported worldwide causes of neonatal mortality to be prematurity and complications in 35% (1 million deaths), intrapartum complications in 24% (0.7 million deaths) and sepsis in 15% (0.6 million deaths) (5).

Korkmaz et al. (26) reported that 76% of all infant deaths occurred in the neonatal period and that the main causes of all neonatal deaths were prematurity and RDS, according to Turkish Ministry of Health Infant Deaths Registry in 2008.

Demirel et al. (28) reported the first 4 ranks of early neonatal deaths as prematurity (47.2%), congenital anomalies (17.5%), infections (6.5%) and perinatal asphyxia (6.1%) according to the neonatal death reports of the Turkish Ministry of Health in 2009.

Late referral of foetuses with congenital anomalies and especially with congenital heart defects to our university hospital which is a major cardiac surgery and genetics reference centre and decreasing incidence of prematurity-related complications, infections and asphyxia are responsible for the proportional increase of congenital anomalies as a reason of perinatal/neonatal mortality.

The limitations of this study are the retrospective nature of data for the former two time points, making statistical comparisons difficult. However, this study highlights ongoing problems regarding antenatal care with foetal deaths and congenital anomalies, while there are important improvements in neonatal care. Neonatal mortality declined and also the major causes of neonatal deaths changed in the last decades.

In conclusion, the high perinatal mortality found in this study is due to high foetal death rate in our regional reference centre, although the NMR is lower than national statistics. All precautions, such as reducing the rate of consanguineous marriages to reduce the incidence of congenital anomalies, better antenatal care, early diagnosis and treatment for high risk pregnancies, must be urgently undertaken in order to prevent foetal deaths.

## Figures and Tables

**Table 1:**
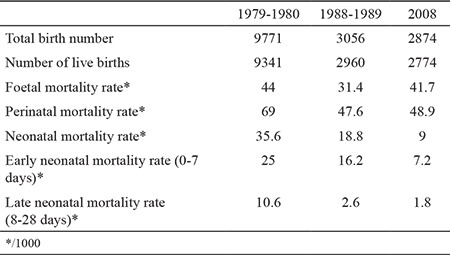
The birth and death statistics in study time points

**Table 2:**
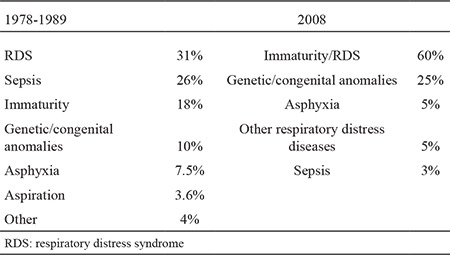
The most common clinical reasons for neonatal mortality
